# Unconscious deception detection measured by finger skin temperature and indirect veracity judgments—results of a registered report

**DOI:** 10.3389/fpsyg.2015.00672

**Published:** 2015-06-08

**Authors:** Anna E. van ’t Veer, Marcello Gallucci, Mariëlle Stel, Ilja van Beest

**Affiliations:** ^1^Department of Social Psychology, Tilburg Institute for Behavioral Economics Research, Tilburg University, Tilburg, Netherlands; ^2^Department of Psychology, University of Milano-Bicocca, Milan, Italy

**Keywords:** deception detection, physiological markers, skin temperature, indirect deception detection, interpersonal relations, psychophysiology

## Abstract

A pre-registered experiment was conducted to examine psychophysiological responses to being lied to. Bridging research on social cognition and deception detection, we hypothesized that observing a liar compared to a truth-teller would decrease finger skin temperature of observers. Participants first watched two targets while not forewarned that they would later be asked to judge (direct and indirect) veracity, and then watched another two targets while forewarned about this. During both these phases finger skin temperature was measured. Findings pertaining to temperature partly confirmed our main hypothesis. When participants were observing a liar, irrespective of being forewarned, on average finger skin temperature declined over time. In the forewarned phase, temperature trajectories of truth-tellers were higher than those of liars, however, in the not forewarned phase, this pattern was reversed. Results confirmed our further hypotheses that participants judge liars as less likeable and less trustworthy than truth-tellers—an indication of indirect deception detection. Our hypothesis that the effect size for trustworthiness would be bigger than that of liking was not supported by the data. Additionally, and also confirming our hypothesis, participants performed around chance level when directly judging whether the target person was lying. Exploratory analyses are reported with regard to truth bias and dependency between direct and indirect veracity judgments. Limitations and directions for future work related to the existence of psychophysiological indicators of deception detection are discussed.

## Results of a Registered Report

The current paper reports the results of research that was conducted after its pre-registration went through peer review and was awarded *In Principle Acceptance*. A methods paper holding the pre-registration of the current research (hereafter referred to as the registered report, RR), including the hypotheses, analysis plan, and proposed sample, as well as a pilot study, can be found online in this special issue (see van ’t Veer et al., 2014). Below we briefly summarize the theoretical background and our hypotheses.

## Brief Summary of Theoretical Background

Being able to detect deception of others—or at the very least knowing whom to trust—was most likely an indispensable advantage during human evolution. Indeed, there are many indications that judging (moral) character and forming impressions of the intentions of others is an elementary, innate ability (e.g., Willis and Todorov, 2006; Fiske et al., 2007; Miller, 2007). Nevertheless, a robust finding in the deception detection literature indicates that people are no better than chance at detecting a liar (Bond and DePaulo, 2006). This is the case, at least, when deception detection ability is assessed with veracity judgments that directly ask about the untruthfulness of a statement. At the same time, an increasing amount of evidence is emerging from the deception detection literature that suggests that people’s judgments of liars and truth-tellers *do* sometimes differentiate between the two—especially when these judgments are assessed in an indirect way.

Evidence for these seemingly more accurate intuitions about the dishonesty of others comes from studies that find *indirect* veracity judgments (i.e., judgments not explicitly aimed at deciding whether deception took place) differentiate between liars and truth-tellers better than *direct* veracity judgments (Vrij et al., 2001; Ulatowska, 2010, 2014). Meta-analyses report that people have more confidence in their judgments after seeing a truth-teller (DePaulo et al., 1997), supporting the idea that feelings of confidence—as indirect measures of deception detection—might differentiate truths from lies. Moreover, subjective, quick, and automatic judgments all seem to be better at distinguishing liars from truth-tellers than their objective, slow, and deliberative counterparts (DePaulo et al., 2003; Albrechtsen et al., 2009).

We suggest that the above-mentioned work can be complimented by not only measuring indirect and direct veracity judgments, but also by exploring the possibility of a physiological marker (i.e., an unconscious indicator) of this indirect deception detection. Building on classical (Harlow, 1958; Bowlby, 1969) as well as more recent work (IJzerman and Koole, 2011; Kang et al., 2011; Szymkow et al., 2013) that relates temperature to trust and perceptions of trustworthiness, we sought to test whether picking up on the deception of others might reflect itself in skin temperature. In the current research, we measure finger skin temperature of people who observe both liars and truth-tellers, as we believe that this physiological proxy of social interaction could be an important indicator of people’s correct intuition toward liars. Previous findings indicate an association between negative or stressful experiences and the lowering of skin temperature (Boudewyns, 1976; Rimm-Kaufman and Kagan, 1996; Kistler et al., 1998; Zhong and Leonardelli, 2008; IJzerman et al., 2012). Therefore, the central hypothesis that we aim to test is whether finger skin temperature will decrease over time when observing a liar compared to when observing a truth-teller.

## Pre-Registered Hypotheses

There were several hypotheses put forward in the pre-registration. To facilitate and clarify the distinction between our confirmatory and exploratory analyses, we restate the hypotheses here. Our main hypothesis was that finger skin temperature would decrease during the watching of a 3-min video clip of a liar (H1). We further hypothesized that participants would judge truth-tellers more trustworthy and likeable than liars (the *indirect* veracity judgments; H2a), with the additional hypothesis that this effect would be bigger for the trustworthiness judgment than for the liking judgment, because trustworthiness judgments are suggested to be more automatic and intuitive and would therefore tap into the covert differences between liars and truth-tellers better (H2b). Next to this, we hypothesized that when asked to judge whether a target person is lying, participants’ judgment would not differentiate between liars and truth-tellers better than chance (the *direct* veracity judgment; H3). Finally, we hypothesized that the indirect veracity judgments, namely the liking and trustworthiness for the target person, would be positively related to finger skin temperature, whereas the direct veracity judgment would not be (H4).

We also included two distinct phases in our experiment. First, participants were *not* forewarned they might be lied to, and subsequently, they *were* forewarned of this possibility. This allowed us to explore whether the hypothesized effects interact with the level of suspicion participants may have. People have their own ideas about what a liar could look like, yet these beliefs about cues are often incorrect (Vrij and Semin, 1996). Having a goal to detect deception could therefore arguably make participants look for these cues more. Additionally, looking for specific cues (e.g., cues that indicate untrustworthiness) may prompt participants to process information more systematically. On the one hand, it could be expected that increased suspicion in the forewarned phase may result in an overall tendency to trust less, without making veracity judgments more accurate (De Neys et al., 2013). On the other hand, being forewarned could benefit the impressions that are formed of targets. Signs of untrustworthiness may more readily be perceived as such due to high accessibility. We therefore tested whether skin temperature, as well as both the direct and the indirect veracity judgment, were differently affected in these two phases.

## Exploratory Research Questions

In addition to the pre-registered hypotheses and exploratory examination mentioned above, we explored our data on the basis of two considerations that occurred after the results were in. First of all, we tested whether on the direct veracity judgment participants were better than chance at detecting deception while controlling for the indirect judgments. We did this to understand the interdependence of the direct and indirect veracity judgments: Although trustworthiness and likeability judgments were counterbalanced, the direct veracity judgment always came after these two indirect judgments. Secondly, we also explored whether participants’ tendency to judge a target as a truth-teller (truth bias) was lower in the forewarned phase than in the not forewarned phase. Truth bias has been argued to be especially prominent during automatic compared to systematic processing (Masip et al., 2009). Therefore, if truth bias were lessened in the forewarned phase this could be an indication, although not conclusively so, of more systematic processing.

## Materials and Methods

### Participants

In accordance^1^ with the RR, data was collected over a period of 3 weeks. Participants received either course credit or €8. This resulted in a total *N* of 191, exceeding our minimal planned sample size of 120 due to running full weeks. We excluded 36 (18.85%) of the participants on the basis of one or more of our predefined exclusion criteria; two participants for knowing that the experiment was about measuring temperature, 28 for being acquainted with one or more of the target people on the videos, 10 for technical failure of the temperature measurement, and one person for smoking more than 20 cigarettes a day. We did not disregard any data points on the basis of our predefined temperature cut-off: participants’ finger skin temperature did not fall below 18° C or above 37° C. Our final sample therefore consisted of 155 participants, 60.65% female, *M*_age_ = 21.35, *SD*_age_ = 3.78, age range: 18–53 years. Participants completed an hour of experiments of which this study was the last half hour, allowing skin temperature to reach a stable level before the experiment began. We did not deviate from the registered minimum sample size, data exclusions, manipulations, or measures in the study except for one instance that we outline in footnote 4.

### Design and Procedure

Participants’ finger skin temperature was measured with an iButton (see Pouw et al., 2012, for software instructions) during the entire experiment. The first experimental factor, veracity of the target person, was manipulated by showing participants a total of four videos containing either a truth-teller or a liar. The second experimental factor, being forewarned or not, was manipulated by not informing participants that the goal of watching the videos was to detect deception for the first two videos (not forewarned phase). For the last two videos, participants were informed of this (forewarned phase)^2^.

All participants first watched a nature documentary of 8 min, which allowed the iButton to reach a stable finger temperature. Participants then watched two videos of 3 min that randomly contained a target person being truthful or untruthful about their identity (not forewarned phase). Next, participants completed our three main dependent variables: for the first and second target person separately, participants were asked to indicate how much they liked this person and how trustworthy they thought this person was (the indirect veracity judgments; both on 7-point scales, order counterbalanced between participants), followed by whether they thought the target person was telling the truth (the direct veracity judgment; forced choice between *yes* or *no*).

We refer to the next phase as the forewarned phase. Due to completing the three main dependent variables participants were now warned about the type of questions they would be asked. From these questions, in turn, they could infer that there was a possibility that the target person would lie. We further stressed the purpose of watching the next videos by telling participants they would get the same questions for these videos. Additionally, all three questions were repeated to help remind them. Participants then proceeded to watch the last two videos that randomly contained a truth-teller or a liar, and completed the three main dependent variables for this third and fourth target person. At the end of the experiment, participants indicated their age, gender, smoking behavior, acquaintance with any of the people presented in the videos, dominant hand, and their thoughts on what the experiment was about.

## Results

All data pre-processing steps (as described in the RR) are available from the first author on request. De-identified data, syntax, R scripts and supplemental material are available online (see https://osf.io/j8w4i/).

### Confirmatory Analyses

#### Temperature Trajectories

**FIGURE 1:**
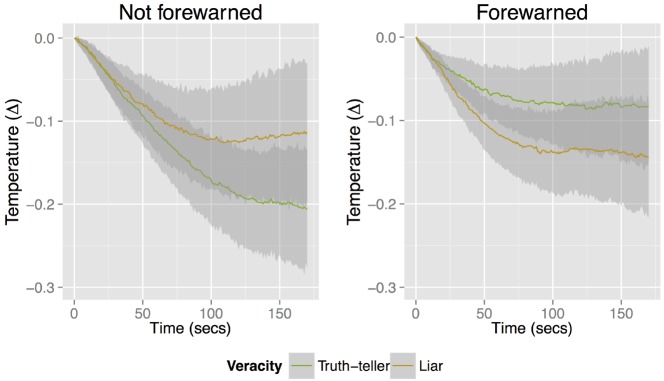
**Average observed temperature change (with confidence interval) over time as a function of veracity of the target person (truth or lie), broken down by phase: While not forewarned of the possibility of deception (left) and while forewarned of the possibility of deception (right)**.

The first hypothesis stated that finger skin temperature would decrease while observing a liar (H1). Figure 1 shows the average temperature trajectories over time for both the not forewarned and forewarned phase. To test the first hypothesis, a model was run in which all factors were modeled as both fixed and random effects in order to estimate the main effect of veracity, the main effect of gender, the main effect of being forewarned, the main effect of order, their interactions, and the interaction of the experimental factors with time. As described in the RR, the interaction of the experimental factors veracity, being forewarned and time informs on whether temperature trajectories change depending on the target person’s veracity and on whether participants were forewarned of the fact that this is a situation in which they have to detect deception. Therefore, the most important effect of the complete model is the interaction effect of the experimental factors and their interaction with time, because the expected change in temperature due to the experimental factors should unfold over time^3^.

There was a main effect of time [*b* = 0.0006, *F*(1,153.026) = 5.251, *p* = 0.023] and a quadratic effect of time [*b* = 0.000007, *F*(1,154.432) = 46.779, *p* < 0.001], indicating that participants’ finger skin temperature dropped during a video and climbed a little toward the end of the video. To grasp the meaning of the size of these effects of time, one could consider that on average our participants’ finger skin temperature was getting one tenth of a degree colder in 3 min. There was also a main effect of gender [*b* = –0.146, *F*(1,154.140) = 6.829, *p* = 0.010] indicating females were relatively colder than males, but there was no interaction of gender with the other experimental factors (all *p*s > 0.341). Neither veracity nor being forewarned had a main effect (*p*s 0.866 and 0.509, respectively), and time did not interact significantly with veracity or being forewarned (*p* = 0.598 and 0.122, respectively). The order of seeing a truth-teller or a liar first did not have an effect on participants’ temperature, nor did the order of judging trustworthiness or liking first (*p* = 0.800 and *p* = 0.848, respectively). Neither of these orders interacted with veracity or being forewarned (*p* = 0.626 and *p* = 0.494, respectively).^4^^,^^5^^,^^6^

Importantly, there was a marginally significant three-way interaction between veracity, being forewarned, and time, *b* = –0.001, *F*(1,154.001) = 3.598, *p* = 0.060, and, also a marginally significant interaction between veracity and being forewarned, *b* = –0.165, *F*(1,153.534) = 3.461, *p* = 0.065 (see Figure 1). Together, these interactions suggests that when participants were not forewarned, their finger skin temperature lowered more when they were watching a truth-teller compared to when they were watching a liar. Yet when participants were forewarned, their finger skin temperature lowered more when they were watching a liar compared to when they were watching a truth-teller. At the end of the videos, for not forewarned participants, watching a truth-teller meant a finger temperature 0.101° C colder than when watching a liar, whereas when participants were forewarned, watching a truth-teller meant a finger temperature 0.077° C warmer than when watching a liar. In other words, when watching truth-tellers, without consciously knowing what they were looking for, our participants’ temperature lowered more than when participants did know what they were looking for. When watching liars, however, temperature lowered no matter whether participants were forewarned or not.

From the marginally significant three-way interaction, we can tentatively conclude that our prediction that finger skin temperature would decrease while participants watch a liar (H1) is supported by the data. However, only for the phase in which participants were forewarned that they could be lied to was this decrease more pronounced than the decrease we observed when participants were watching truth-tellers. Moreover, there was significant variation at the participant level, meaning that the effect of observing a liar or a truth-teller on skin temperature varied from one participant to another. We should therefore not exclude the possibility that there is an unknown individual difference characteristic that moderates the relationship between veracity and skin temperature (cf., Whitsett and Shoda, 2014).

#### Indirect Veracity Judgments

**FIGURE 2:**
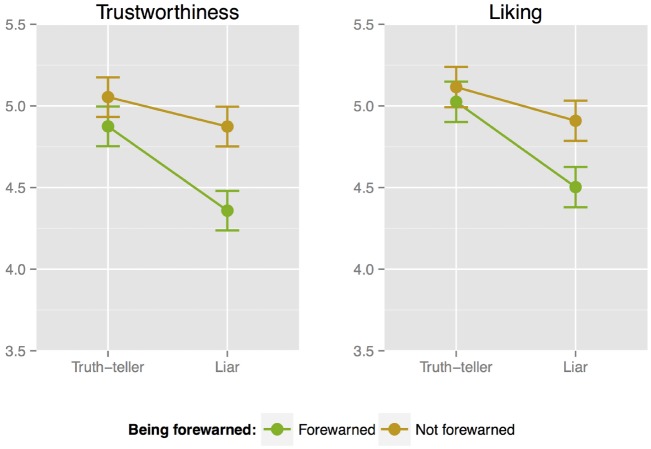
**Means and standard errors of the indirect veracity judgments (trustworthiness and liking) of the target person, for truth-tellers and liars, by phase (not forewarned and forewarned)**.

Figure 2 depicts the means and standard errors of the indirect veracity judgments (trustworthiness and liking). We ran two separate models to test Hypothesis 2a; the first to assess whether liars were liked less than truth-tellers, the second to assess whether liars were rated lower on trustworthiness than truth-tellers.

The first linear mixed model with liking as the dependent variable and veracity, being forewarned, and order as predictors revealed a main effect of veracity, *b* = –0.364, *F*(1,461.532) = 13.735, *p* < 0.001, meaning that on average liars were liked less (*M* = 4.706, *SE* = 0.79) than truth-tellers (*M* = 5.071, *SE* = 0.79). There was also a main effect of being forewarned, *b* = –0.247, *F*(1,462.203) = 6.287, *p* = 0.013, meaning that when participants were forewarned, they judged the target person less likeable. There was no interaction between veracity and being forewarned (*p* = 0.109), and no effect of order of seeing a truth-teller or a liar first (*p* = 0.717). The order of first judging liking of the target person and then judging the trustworthiness of the target person, or *visa versa*, did have an effect, *b* = –0.307, *F*(1,153.792) = 6.012, *p* = 0.015, such that if a participant first judged liking, their liking judgment was higher than if a participant first judged trustworthiness and then liking. Regarding the random intercepts, we found a non-zero variance (σ = 0.226, Wald *Z* = 3.102, *p* = 0.002), implying that participants have different average liking ratings.

The second linear mixed model with trustworthiness as the dependent variable and veracity, being forewarned, and order as predictors revealed an average effect of veracity, *b* = –0.348, *F*(1,461.656) = 11.843, *p* < 0.001, meaning that on average liars were deemed less trustworthy (*M* = 4.616, *SE* = 0.079) than truth-tellers (*M* = 4.965, *SE* = 0.079). There was also a main effect of being forewarned, *b* = –0.347, *F*(1,462.340) = 11.742, *p* < 0.001, meaning that when participants were forewarned, they judged the target person less trustworthy. There was a marginally significant interaction between veracity and being forewarned, *b* = –0.335, *F*(1,461.656) = 2.746, *p* = 0.098, suggesting that the difference on the trustworthiness judgment between liars and truth-tellers became bigger in the forewarned phase. Both the order of seeing a truth-teller or a liar first and the order of the indirect veracity judgments were not significant predictors in this model (*p =* 0.783 and 0.217 respectively). Regarding the random intercepts, we found a non-zero variance (σ = 0.180, Wald *Z* = 2.546, *p* = 0.011).

Taken together, the results of the indirect veracity judgments suggest that liars were liked and trusted less than truth-tellers. It also seems to be the case that being forewarned of the possibility of deceit made participants more distrusting overall, and especially so for liars. This indicates that if participants are alerted to the fact that they can be deceived, this helps them form better impressions of the target person’s sincerity.

Hypothesis H2b stated that the effect of rating truth-tellers more positive than liars on the indirect veracity measures would be bigger for trustworthiness, as trustworthiness judgments are suggested to be automatic and intuitive and could therefore better distinguish the subtle differences between truth-tellers and liars. We tested whether the effect size of veracity on the trustworthiness judgment was higher than the effect size of veracity on the liking judgment. To obtain this test we estimated a mixed model with both trust and liking judgments combined as a single dependent variable, veracity as independent variable, and with appropriate dummies indicating whether the scores refer to trustworthiness or liking. The interaction between the veracity term and the dummy provides the required test. This technique, one of the different ways to estimate a seemingly unrelated equation model, is largely inspired by random coefficients mediational models (MacCallum et al., 1997; Bauer et al., 2006). We found no difference between effect sizes, coefficients difference = 0.019, *t*(1073.8) = 0.130, *p* = 0.896.^7^

#### Direct Veracity Judgment

We estimated a mixed logistic model with participants’ accuracy on the direct veracity judgment (“Is this person telling the truth, yes or no?”), with veracity, being forewarned, and order as predictors. Because the mixed model did not converge, the logistic regression parameters and the associated inferential tests were obtained with GEE methodology (Zeger et al., 1988). An exchangeable working correlation matrix was used to model the dependency of observations. Results showed a main effect of veracity, χ^2^(1) = 99.375, *p* < 0.001, such that the probability of a correct response was higher when participants had been watching a truth-teller compared to a liar. There was no main effect of being forewarned χ^2^(1) = 1.211, *p* = 0.270, and being forewarned and veracity did not interact, χ^2^(1) = 0.40, *p* = 0.841. There was no effect of order (*p* = 0.998).

These results indicate that although participants were more accurate in detecting truth-tellers—a finding probably due to truth-bias (see also under exploratory analyses)—being forewarned or not did not significantly affect the ability to correctly detect liars and truth-tellers. Despite of this, there is value in examining whether for the different phases the probability of correctly detecting deception is higher than chance (H3). To test the accuracy of participants direct veracity judgments against chance in the two phases, we ran the same model as described above, with now only being forewarned as the independent variable. In the not forewarned phase, participants were correct 52.92% of the time, which did not differ from chance, χ^2^(1) = 0.725, *p* = 0.395, Wald 95% CI [0.47, 0.58]. In the forewarned phase, participants were correct 57.42% of the time, which was significantly different from chance, χ^2^(1) = 9.704, *p* = 0.002, Wald 95% CI [0.53, 0.63]. We further examined whether this higher accuracy rate in the forewarned phase could be explained by the indirect veracity judgments that were assessed right before it. We did this because the results also indicated that the indirect veracity judgments were affected by being forewarned. We report on this analysis under exploratory analyses.

#### Relationship between Temperature and the Veracity Judgments

To assess whether participants’ temperature at the end of the videos is predictive of their indirect and direct veracity judgments we ran separate models with temperature as the independent and liking, trustworthiness, accuracy of participants answer on the direct veracity judgment, and the answer of the direct veracity judgment as dependent variables. We did not find a relationship between temperature and any of these self-report measures. Temperature was not predictive of liking, *F*(1,610.689) = 1.661, *p* = 0.198, and it did not interact with the experimental factors to predict liking, all *p*s > 0.137. Temperature was also not predictive of the trustworthiness judgment, *F*(1,609.964) = 2.358, *p* = 0.125. The interaction of being forewarned and temperature on trustworthiness was marginally significant (*p* = 0.085), however, interactions of veracity with temperature as well as the interaction of veracity with being forewarned and temperature were not significant (all *p*s > 0.633). For the direct veracity judgment, temperature did not predict whether the direct veracity judgment was correct, χ^2^(1) = 0.009, *p* = 0.923, nor did it predict whether participants said yes or no to the question whether the target person was telling the truth, χ^2^(1) = 0.757, *p* = 0.384.

### Exploratory Analyses

#### Detection Accuracy When Controlling for Indirect Judgments

As is described above, we found participants’ accuracy in detecting deception in the forewarned phase to be significantly higher than can be expected by chance. We also found an effect of being forewarned on the trustworthiness judgment, such that in the forewarned phase, participants were more likely to judge a liar as less trustworthy. It could be the case, therefore, that because the indirect veracity judgments (i.e., liking and trustworthiness) were judged before the direct veracity judgment, participants accuracy on the direct veracity judgments was enhanced due to an enhanced performance on the indirect measures. To examine this possibility, we ran the model assessing whether accuracy at detecting deception was better than chance again, this time controlling for the indirect veracity judgments. Comparable as to when not controlling for these judgments, participants’ deception detection accuracy was not significantly different from chance in the not forewarned phase χ^2^(1) = 0.576, *p* = 0.448, Wald 95% CI [0.34, 0.71]. However, as a result of controlling for these judgments, in the forewarned phase, participants’ accuracy was no longer significantly different from chance either, χ^2^(1) = 1.982, *p* = 0.159, Wald 95% CI [0.39, 0.75]. This could imply a reliance of the direct veracity judgment on the indirect measures, although we are hesitant to make any firm conclusions on the basis of these results.

#### Truth Bias

The finding that in the forewarned phase participants seemed to be better able to distinguish between liars and truth-tellers on both the indirect as well as the direct veracity judgment could be a side effect of an overall change in the tendency to judge messages as true. Our manipulation of being forewarned could have made participants more suspicious overall, leading them to judge a lower proportion of messages as truths. To test whether this was the case, we ran a GEE with the tendency to judge a message as the truth as the dependent variable and being forewarned and veracity as the independent variables. Although the tendency to make truth judgments was higher when actually watching a truth-teller compared to a liar, χ^2^(1) = 7.962, *p* < 0.01, the tendency to make truth judgments did not differ between the different phases, χ^2^(1) = 0.612, *p* = 0.434. Participants thus did not differ in their amount of truth judgments when watching truths in the not forewarned phase (proportion = 0.733) and the forewarned phase (proportion = 0.781), and they did not differ in their amount of truth judgments when watching lies in the not forewarned phase (proportion = 0.675) and the forewarned phase (proportion = 0.632).

## Discussion

With this registered study we explored people’s automatic evaluative and physiological responses to observing a deceiver, as well as their more conscious direct evaluation of a target person’s veracity. We did so in two distinct phases: First while participants were not forewarned of what the goal of observing another person was, and second while participants were forewarned that the goal was to form an impression of this other person’s likeability, trustworthiness, and their veracity. We chose to measure participants’ finger skin temperature because of the suggested embodied function of warmth in interpersonal relationships. The observed patterns of temperature change over time only partly confirmed our main hypothesis (H1), and the current findings pertaining to this hypothesis are therefore inconclusive. We found that finger skin temperature consistently decreased while observing a liar. When participants were observing a truth-teller, however, their finger skin temperature decreased more than it did for liars in the phase where participants did not have the goal to detect deception. In contrast, participant’s finger skin temperature stayed higher when observing a truth-teller compared to a liar when participants did have the goal to detect deception. This latter pattern is consistent with the direction of our predictions based on the relationship between warmth and positive person impressions; however, it failed to achieve significance by conventional standards.

As for the judgments of trustworthiness and likeability—the so-called indirect veracity judgments—we found that across both phases, liars were consistently judged less trustworthy and less likeable than truth-tellers. This is in accordance with our hypothesis (H2a) which was based on earlier findings in the literature indicating that subjective, indirect, and intuitive judgments can contrast liars from truth-tellers (Vrij et al., 2001; DePaulo et al., 2003; Albrechtsen et al., 2009) and this finding replicates our earlier findings of the pilot study described in the RR (see van ’t Veer et al., 2014) associated with the current study. In addition, results indicated that in the forewarned phase participants were more inhibited in their liking and trusting overall. Irrespective of this, trustworthiness and liking judgments were lower for liars than for truth-tellers in both phases. It thus seems that intuitive judgments are reliable guides when forming impressions of the intentions of others no matter whether people are forewarned or not.

We did not observe a difference between the magnitude of the effect of the likeability judgment and the trustworthiness judgment. This fails to support our hypothesis that trustworthiness judgments would differentiate between liars and truth-tellers better than likeability judgments because of the relative automaticity that has been argued to underlie trustworthiness impression formation (H2b). In the current study the order of these two indirect veracity judgments was counterbalanced. For the likeability judgment we found the order of the questions to influence the judgments themselves: The target person was judged less likeable if trustworthiness was assessed first. These judgments were also highly correlated, suggesting that we cannot draw far-reaching conclusions from the fact that we found similar magnitudes of effects. Future research may test the difference in strength of these judgments in a between participants design rather than a within design.

For people’s ability to accurately indicate whether someone was lying, we predicted that this direct veracity judgment would not detect deception much better than chance (H3), as a meta-analysis by Bond and DePaulo (2006) would suggest. We indeed found participants to be accurate about 53% of the time in the not forewarned phase, a performance that was not different from chance. However, when participants were alerted to the possibility of deceit and had the goal to detect deception, their accuracy rose to 57%. This percentage was significantly different from chance, although not as substantial as to suggest our participants were able to catch liars with a high success rate. It seems that, at least in the case where participants are searching for indications of ill intend, they had a slightly higher chance of correctly detecting a liar. This can be the case, for instance, because of a reduced truth-bias under conditions where people are more suspicious, or, as our exploratory analyses suggest, because intuitive impressions of others are aided by being a bit more on guard. A combination of these two processes could also be at play: Adjustment of the automatic tendency to judge most people to be evidently honest for the perception of ill-intent could require motivational resources (i.e., the goal to detect deception) as well as indications from indirectly formed impressions. Future research could examine whether it best to be on guard while relying on intuition at the same time. We suggest that it is likely that this is what happens when affective judgments are made in a context in which deception is more salient.

Taken together, our participants’ accuracy in correctly indicating whether they were being lied to was around chance level, and their impressions of liars’ likeability and trustworthiness were likely to be more negative than their impressions of truth-tellers. Although comparing participants’ accuracy on the direct judgment to the indirect judgments is not a fair comparison in this case, in the pilot study we described in the RR, we assessed both types of judgments on a continuous scale, allowing for a better comparison. Results of the pilot study indicated very clearly that participants did not explicitly judge liars to be more deceptive than truth-tellers. In contrast to this, the indirect judgment that assessed to what extent a target person was likeable was significantly lower for liars compared to truth-tellers. Similar evidence for the superior accuracy of indirect questions has been found by others, for instance when using the question how hard a target person was thinking compared to the question whether the target person was lying (Ulatowska, 2014). Our results further imply that one and the same person is judged more negatively when he or she is lying compared to when telling the truth, even though this person has a good chance of ostensibly getting away with giving off a false impression.

With regard to the relationship between our self-report measures and skin temperature (H4), we found no meaningful correlations. This can be contrasted with the results of the pilot study, where we did observe a positive correlation between (a) temperature and liking the target person and (b) temperature and judging the target person to be telling the truth. This discrepancy calls for further exploration of the functioning of thermoregulation in response to real life social interactions and the possible interplay of physiological and psychological processes during deception detection.

As our main findings pertaining to the temperature measure reached marginal significance, we are hesitant to draw firm conclusions, and suggest future explorations in this area to consider an even bigger sample size. Currently, we found an initial tentative trace of the supposition that observing a deceiver can influence the physiology of the observer. More specifically, our findings hint at a thermoregulatory mechanism that responds to the veracity of an impression another person is trying to convey.

### Being Forewarned of The Possibility of Deceit

There are several possible explanations for why being forewarned or not would lead to different processes and outcomes. Below we discuss these explanations and relate them to our findings for the direct and indirect veracity judgments, the physiological changes and our previous findings in the pilot study described in the RR. Our data suggest liars have more chance of getting caught when their observer is alerted to the possibility of deceit. This indicates that detection—and possibly the ability to process the rich variety of information that is sent by the target person—is aided by having a detection goal while decoding a message. It has been previously argued that having correct beliefs about what cues give a liar away benefits the observer only when such beliefs are activated while making the judgment: People have been found to be better at detecting a liar when they are both told a target is “usually untruthful” (thus creating suspicion) and rely on accurate non-verbal cues to deception (Forrest et al., 2004). A possible explanation thus seems to be that some level of active engagement in detection is beneficial because distinct processes are switched on.

Our indirect measures seemed to be slightly more powerful in differentiating liars from truth-tellers when participants were forewarned. One possible explanation that has been provided for the finding that indirect questions are more accurate than direct questions is that the indirect questions shift participants’ attention to the appropriate cues to deception (Vrij et al., 2001). However, this explanation does not seem to account for sharpened differentiation when the aim to detect deception is salient; indirect questions have been found to discriminate between liars and truth-tellers even though participants were not informed about the reason for why they were being assessed (Ulatowska, 2010, 2014). In the current experiment, we chose to measure person impressions related to warmth because these judgments are more intuitive compared to other indirect measures (e.g., whether the target person had to think hard). In contrast to these other indirect measures, the liking and trustworthiness judgments seem unlikely to shift attention to specific cues that indicate deception. Instead, they elicit a more holistic and affective evaluation. Relying on specific cues could be costly when beliefs about the characteristics of deceptive behavior are not correct (Forrest et al., 2004). Holistic judgments, in comparison, have more chance of getting it right if intuition based on the rich information send by liars indeed aids deception detection. This is further suggested by studies showing that task-relevant unconscious thought improves lie detection (Reinhard et al., 2013). Indeed, our indirect judgments proved appropriate guides to trustworthiness even when participants were arguably not searching for cues of deception (i.e., in the not forewarned phase). We encourage future research to further test whether these holistic indirect judgments perform better when the goal to detect deception is present.

Most research on deception detection has explicitly given participants the instructions to watch a video with the goal of detecting deception (Reinhard et al., 2013). It seems, however, that this does not mimic real life situations in which people are usually not out to spot liars—notwithstanding the notable exception of law enforcement professionals. Even so, under these explicit instructions to detect deception, intuitive judgments seem to outperform deliberative ones (cf. Albrechtsen et al., 2009). Our experiment, in contrast to other experiments, was characterized by two distinct phases: One where participants watched videos while the reason for this was unbeknownst to them, and one where the goal of watching videos was apparent. This allowed us to explore differences our physiological measure for these two phases. While we had suggested that observing a liar would result in a lower skin temperature than observing a truth-teller, this was only the case when participants had a clear goal: to detect deception.

One possible explanation for this could be that while people generally go through life unsuspicious of others, when they have the goal to detect “threats” in the environment their conscious as well as their unconscious reactions are conducted to respond to this threat more adaptively. One could imagine, for instance, that being more vigilant heightens conscious processing of information while at the same time it increases the reflexive, automatic responses. It has been argued that some automatic processes are goal-dependent and require awareness of the triggering stimulus to occur (Bargh, 1994), and that external stimuli and internal determinants of behavior are mutually dependent on each other in producing adaptive responses (Fiedler et al., 2009). For instance, unintentional, spontaneous trait inferences happen with little awareness, yet they are goal-dependent in the sense that they arise when prompted by a relevant goal (Fiske and Taylor, 2013). It could be the case that similar processes were unintentionally elicited in our experiment due to giving participants the explicit instruction to form impressions of possible deceivers. Future research could examine this possibility by exploring the relationship between physiological responses and having, versus not having, a conscious goal.

When comparing the current results to the results obtained in the pilot study, the temperature pattern observed in the second phase of the pilot study seems to resemble the current pattern observed in the not forewarned phase. Although speculative, a perceivable cause of this could be that in the pilot study the forewarning was not manipulated as strong as in the current study, leaving participants still in a relatively ignorant state about what was to come and whether the experimental context was one of deception detection. Being able to expect and prepare for what is to come arguably has some advantages, although it should be noted that not anticipating threats is comparable to an everyday life situation in which people assume they will not be lied to.

### Limitations and Directions for Future Research

In the current study, detection accuracy was slightly higher than can be expected by chance in forewarned phase, even though truth-bias was unaltered. This means that participants’ judgments in the forewarned phase were less often false alarms and more often hits. This could be due to a learning effect, although this seems unlikely. Our design was set up to minimize the possibility of participants getting better over time; participants did not get feedback on their performance, videos were randomized, and the procedure of seeing a video and answering questions about it was “rehearsed” with the nature documentary. We cannot, however, exclude the possibility that the differences between the two phases stems from the time participants were on the task, as this feature is inherent to our within-design. Similarly, we cannot exclude the possibility that the accuracy of the direct veracity judgment was assisted by the mere presence of both the indirect veracity judgments that came before it.

Next to these design characteristics, another possible explanation for the differences between our experimental phases could be that simply having thought and read about lying could make this concept more accessible, unintentionally influencing inferences and impressions of the target person to come. An intuition about a person that is based on experiential, associative knowledge might be triggered by deliberate thought (cf. Epstein, 2003). A relevant question for future research is whether activating knowledge structures concerning distrust would lead to similar enhancement of (indirect) deception detection as we found here.

In our experiment we found liars to be liked and trusted less. A possible explanation for this effect of indirect deception detection is that liars come across more tense and may exhibit afflicting emotions related to lying, which could lead to emotional contagion. We did not ask our participants whether they themselves felt tense after seeing a liar; however, it could be argued that the temperature measurement is a proxy for this. Future research will have to identify the exact relationship between so-called indirect veracity judgments and other measures indicative of the affective state of the observer. Although emotional contagion from the liar to the observer seems plausible, on the basis of what is currently known, it is too soon to draw any conclusions. Studies where participants were asked how comfortable they felt after a deceptive message reveal contradicting patterns (DePaulo and Morris, 2004; Ulatowska, 2014), possibly due to features of the sender such as whether their task of lying was cognitively demanding or the extent to which they themselves feel comfortable lying. If contagion is indeed present, a possible prediction could be that the negative feelings elicited in the observer would aid affective judgments. However, when it comes to consciously catching the liar these feelings may induce more systematic processing and therefore hinder direct detection of veracity.

The fact that our experimental design was able to elicit changes in temperature is promising, especially in light of the fact that the use of videos to manipulate truths and lies is a minimal, albeit controlled, version of real interactions. In general, deception detection performance is equally poor when observers detect deception in a live situation compared to observing a video (for an overview, see: Landström et al., 2005). It is likely, however, that a target person’s deceptive intent is registered as a threat to a lesser extend when presented on a video than in a real life interaction. It is also conceivable that this low level of threat might need some higher alertness or vigilance to be detected, as could be the case when being forewarned. Manipulations that aim to make the environment more unpredictable would in that case enhance the ability to detect untrustworthiness. Additionally, people who are constantly more on guard and distrusting (e.g., insecurely attached individuals) may see more deception around them. Whether they are more often accurate in these assessments remains an unanswered question. Furthermore, it may well be the case that interacting with a liar in real life is costly and aversive because the observer needs to be more on the alert. To explore this possibility and to make a broad generalization possible, more data from accumulating accounts based on different sets of videos and real life interactions is needed.

To examine the impact of deceptive messages on the observer further, more time-sensitive methods would shed light on the dynamic interplay between interaction partners and its assumingly adaptive nature. For instance, neural activity associated with observing a deceptive message could be considered, as well as mental activity as assessed by pupillary responses. Other areas of investigation involve the long-term consequences of insincere interactions on, for instance, judgments of moral character.

## Conclusion

The current research is a first endeavor to explore psychophysiological underpinnings of deception detection with a special focus on thermoregulation within the observer of truthful and deceptive messages. We found marginal significant results revealing skin temperature decreased when liars were observed, whereas temperature trajectories for observing truth-tellers were dependent on being forewarned of the possibility of deceit. Indirect judgments of liars and truth-tellers revealed that lying typically caused a person to be liked and trusted less, while accuracy on a direct judgment of whether this person was lying was barely above chance level.

## Pre-Registration

The aim of this registered experiment was to investigate a previously unexplored part of deceptive social interactions and the role of psychophysiology (i.e., the embodiment) of these interactions on the part of the receiving end of a deceptive message. This research, fundamental in its nature, is but an example of the multitude of opportunities for further investigation—some of which we have suggested above. The fact that this research was pre-registered played a positive role in the development of our experiment and it did not constrain our curiosity for exploring the data. Working together with reviewers in an early stage maximizes chances of making a valuable contribution to current debates. In addition, this process and its open access character carry on the momentum, enabling researchers to continuously build on ongoing work. We hope to have inspired future investigations of the interaction between physiology and cognition, and the possible influences of this interaction for social relationships.

### Conflict of Interest Statement

The Associate Editor Hans IJzerman declares that, despite having collaborated with authors Ilja van Beest and Marcello Gallucci, the review process was handled objectively and no conflict of interest exists. The authors declare that the research was conducted in the absence of any commercial or financial relationships that could be construed as a potential conflict of interest.

## Footnotes

1 For a description of adequate detail to allow close replication we refer to the pre-registration of this experiment (van ’t Veer et al., 2014).

2 In the RR these two phases were termed “unaware” and “aware.”

3 As planned, we computed participants’ temperature minus their temperature at the beginning of the video, and because we should allow some time for any influence of our manipulations to take effect, we centered time on the last second of the video. This deviates from our original plan to center time on 85 s, however it does not influence the interactions between time, veracity, and being forewarned that are of interest. This will produce an estimate (*b*) of the influence of observing a truth-teller or a liar for approximately 3 min on participants’ finger skin temperature.

4 In the RR we stated that participants who had “prior experience with the temperature measure (and its debriefing) will be offered participation in another study and will be refused participation in the current study on theoretical grounds, as people may be able to consciously control their own finger temperature (Keefe, 1978).” Two participants did participate but stated in their comments they were aware that temperature was measured, and thus we saw fit to exclude them. Not excluding these two participants does not change the overall results: The interaction between veracity and being forewarned is still marginally significant, *b* = –0.173, *F*(1,155.599) = 3.758, *p* = 0.054 and so is the interaction between veracity, being forewarned, and time, *b* = –0.001, *F*(1,156.000) = 3.780, *p* = 0.054.

5 We excluded one participant who indicated to smoke more than 20 cigarettes a day (as our pre-defined exclusion criteria required). Including this participant did not change any of the interpretations of the results, both the interaction between veracity and being forewarned and the interaction between veracity, being forewarned, and time were marginally significant [*b* = –0.166, *F*(1,154.453) = 3.552, *p* = 0.061, and *b* < 0.001, *F*(1,155.000) = 3.655, *p* = 0.058, respectively].

6 As was specified in the RR, we also ran our full model while excluding participants who did not reach 30° C during the neutral nature movie. This left a total of 108 participants (about two thirds of our total sample; *M*_age_ = 21.49, *SD*_age_ = 4.25, 53.70% female). Excluding these participants did not change the estimate of the veracity × being forewarned interaction, however, the effect did become non-significant: *b* = –0.168, *F*(1,107.780) = 2.259, *p* = 0.136.

7 We also explored the relationship between liking and trustworthiness judgments by using a mixed model with random intercepts after standardizing these variables, and found that liking and trustworthiness judgments were highly correlated (0.670).
